# Photo-assisted electrochemical advanced oxidation processes for the disinfection of aqueous solutions: A review

**DOI:** 10.1016/j.chemosphere.2021.129957

**Published:** 2021-07

**Authors:** Josué Daniel García-Espinoza, Irma Robles, Alfonso Durán-Moreno, Luis A. Godínez

**Affiliations:** a Centro de Investigación y Desarrollo Tecnológico en Electroquímica, Parque Tecnológico Querétaro Sanfandila, 76703, Pedro Escobedo, Querétaro, Mexico; b Facultad de Química, Universidad Nacional Autónoma de México, CDMX, Mexico

**Keywords:** Photo-assisted AOPs, Advanced oxidation processes, Pathogen inactivation, Electrochemical disinfection, Photo-assisted disinfection

## Abstract

Disinfection is usually the final step in water treatment and its effectiveness is of paramount importance in ensuring public health. Chlorination, ultraviolet (UV) irradiation and ozone (O_3_) are currently the most common methods for water disinfection; however, the generation of toxic by-products and the non-remnant effect of UV and O_3_ still constitute major drawbacks. Photo-assisted electrochemical advanced oxidation processes (EAOPs) on the other hand, appear as a potentially effective option for water disinfection. In these processes, the synergism between electrochemically produced active species and photo-generated radicals, improve their performance when compared with the corresponding separate processes and with other physical or chemical approaches. In photo-assisted EAOPs the inactivation of pathogens takes place by means of mechanisms that occur at different distances from the anode, that is: (i) directly at the electrode’s surface (direct oxidation), (ii) at the anode’s vicinity by means of electrochemically generated hydroxyl radical species (quasi-direct), (iii) or at the bulk solution (away from the electrode surface) by photo-electrogenerated active species (indirect oxidation).

This review addresses state of the art reports concerning the inactivation of pathogens in water by means of photo-assisted EAOPs such as photo-electrocatalytic process, photo-assisted electrochemical oxidation, photo-electrocoagulation and cathodic processes. By focusing on the oxidation mechanism, it was found that while quasi-direct oxidation is the preponderant inactivation mechanism, the photo-electrocatalytic process using semiconductor materials is the most studied method as revealed by numerous reports in the literature. Advantages, disadvantages, trends and perspectives for water disinfection in photo-assisted EAOPs are also analyzed in this work.

## Highlights

• There is an increasing potential for photo-assisted EAO disinfection processes.
• Disinfection at the electrode/solution interphase depends on the anode material.
• Indirect disinfection can be carried out by a variety of electro-generated oxidants.
• Solar EAOPs are effective and cost competitive approaches for pathogen inactivation.

## Graphical abstract

**Figure:**
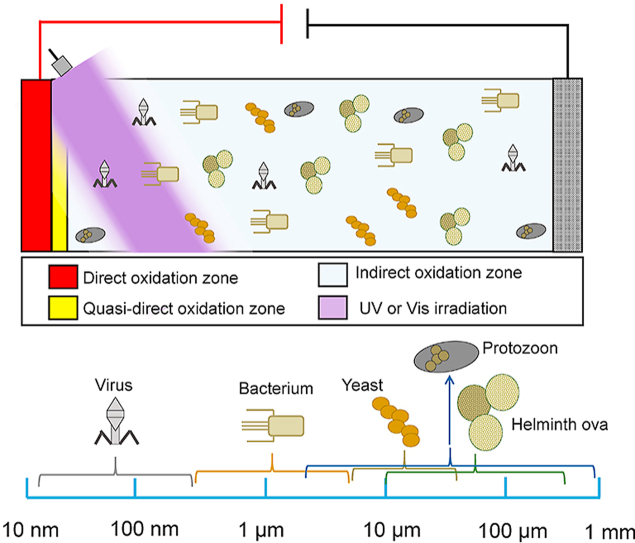

## Abbreviations, acronyms and symbols

AOPs: Advanced oxidation processes
BDD: Boron doped diamond
CFU: Colony forming unit
DOM: Dissolved organic matter
DSA: Dimensionally stable anode
E°: Standard oxidation potential
EAOPs: Electrochemical advanced oxidation processes
EC: Electro-coagulation
FTO: Fluorine-doped tin oxide
GAC: Granular activated carbon
h^+^: holes
*hv*: quantum energy of a photon
λ: wavelength
MMO: Mixed metal oxide
NOM: Natural organic matter
NTN: Nanotubes
PEC: Photo-electrocatalysis
PTFE: Polytetrafluoroethylene
RHS: Reactive halogen species
ROS: Reactive oxygen species
SHE: Standard hydrogen electrode
SS: Stainless-steel
UV: Ultraviolet
WHO: World Health Organization

## 1 Introduction

According to the Update and Sustainable Development Goal Baselines of UNICEF and the World Health Organization (WHO), 2.3 billion people currently lack basic sanitation services and about 159 million people still collect drinking water directly from surface water sources (World Health Organization, 2017). Treated wastewater quality often limits its reuse, mainly in those regions where the infrastructure is not sufficient and therefore, poor treatment and deficient disinfection represents a serious health problem. In this context, it is important to point out that fecal contaminated water sources are particularly troublesome. Diarrhea, for instance, is one of the world’s major killers resulting in approximately 4% of all deaths worldwide (WHO/UNICEF, 2017; World Health Organization, 2017).

Human pathogens, that are transmitted through fecal contamination, are not only common cause for disease in drinking water, but have also been found to persist for considerable periods of time in water sources and in soil which, in time, drains into underground water supplies (Macphee et al., 2009). In addition to fecal source pathogens such as enteric bacteria (e.g., strains of *E. coli*), domestic wastewater is characterized by organic matter, nutrients, pathogens and traces of bio-recalcitrant pollutants. In this regard, it is widely accepted that althought the removal of easily degradable wastewater pollutants such as organic matter, nitrogen and phosphorus is efficiently carried out by means of biological processes, the degradation of persistent pollutants and the disinfection step, often constitute the final and the most relevant stage of the wastewater treatment process (Martínez-Huitle and Brillas, 2008).

Nowadays, chlorination is by far the most popular method for the disinfection of drinking-water and treated wastewater. Chlorine kills harmful microorganisms, has decolorization properties and oxidize most organic molecules (Cheswick et al., 2020; Drogui and Daghrir, 2015; Martínez-Huitle and Brillas, 2008). The disinfection mechanism of free chlorine on bacterial cells relates to oxidative damage to membranes, nucleic acids, proteins, amino acids, cell walls and other lipids, causing a loss of viability (Cheswick et al., 2020). However, the interaction of active chlorine and natural organic matter (NOM) produces disinfection by-products such as trihalomethanes, haloacetic acids, haloacetonitriles and haloketones, some of which are endocrine disruptors involved in brain cancer, immune and reproductive system problems and organism feminization. Therefore, the generation of halogenated by-products in chlorination process represents a considerable disadvantage from the environmental and health points of view (Lacasa et al., 2019).

Ultraviolet (UV) assisted disinfection processes on the other hand, constitute a popular and attractive alternative to chlorination for water reuse, wastewater reclamation and domestic water disinfection (Carré et al., 2018; Gallandat et al., 2021; Li et al., 2020; Zhang et al., 2020a). Although UV treated wastewaters do not contain halogenated by-products, it has been found that several organic compounds and pollutants can be partially degraded under UV irradiation giving rise to by-products that are often more toxic than their parent compounds (Russo et al., 2019). In addition, the disinfection efficiency of UV is affected by several factors such as suspended particles concentration and size or dispersed microbial concentration (He et al., 2020). It is important to point out that some antibiotic resistant bacteria survive after UV disinfection (Guo and Kong, 2019) and some other bacteria revive in the darkness after UV treatment (Chen et al., 2020). Furthermore, a number of pathogens, particularly some virus species, are naturally resistant to traditional treatments such as UV and chlorination (Li et al., 2014).

Ozone (O_3_) is also a strong oxidizing agent and an efficient option for pathogen inactivation (Chang et al., 2020; Girgin Ersoy et al., 2019; Perry et al., 2019) as well as for decolorization, taste and odor control for water reuse (Zhang et al., 2020d). Its performance in disinfection processes of real secondary effluents has not only found to be quite efficient by itself but also substantially improved by synergetic coupling to hydrogen peroxide (H_2_O_2_) or UV irradiation (Malvestiti and Dantas, 2018). Ozone disinfection however is an expensive process without remnant effect that can also be characterized by the formation of toxic by-products (Drogui and Daghrir, 2015). Furthermore, it has some limitations in terms of the inactivation of resistant pathogens such as *Cryptosporidium parvumoocysts* and *Giardia lamblia* (Shi et al., 2021). In addition, competitive reactions with organic matter, as well as changes in pH, alkalinity and temperature, could modify its solubility in water and affect the oxidant efficiency (Meas et al., 2011, Zhang et al., 2020d).

Separation processes such as adsorption using biochars, biosand or geosorbents have also been used for pathogen removal (Guan et al., 2020; Mira Anuar and Chan, 2020; Rahman et al., 2020). In these cases, however, the eventual saturation of the adsorbent surface and the fact that some pathogens cannot be retained due to their large size when compared to the dimensions of the pore and channel structure of the adsorbent, have resulted in a limited use of the adsorption approximation. In this regard, other separation technologies such as ultrafiltration, nanofiltration or reverse osmosis have been developed to efficiently remove pathogens from water (pore size of the membranes of 2 nm) (Cruz et al., 2017; El-Ghzizel et al., 2020). In these cases however, other limitations such as membrane fouling and the high trans membrane pressure (up to 100 bar) are considered as two of the main challenges to overcome in the future in order to increase the effectiveness of this approach (Metcalf and Eddy, 2003).

Despite the uncomplicated and simple performance of these processes, their high chemical consumption and treatment cost constitute major barriers in field applications (Garcia-Segura et al., 2020; Lacasa et al., 2019).

To overcome these drawbacks, the development of alternative disinfection technologies is still an attractive and major focus of research. In this regard, advanced oxidation processes (AOPs) stand out as a feasible approach for the inactivation of pathogens. AOPs are characterized by the generation of highly reactive oxidizing species, particularly hydroxyl radicals (^•^OH) with a high standard oxidation potential (E° = 2.8 V *vs.* standard hydrogen electrode, SHE) that are capable of attacking a wide variety of pollutant species (Brillas, 2020; Martínez-Huitle and Brillas, 2009; Martinez-Huitle and Ferro, 2007).

Several AOPs have been proved for disinfection purposes. For instance, UV/H_2_O_2_ process is five times faster in inactivation and inhibition of microorganisms as well as in degrading aromatic compounds than those of other popular technologies (Bustillo-Lecompte et al., 2016; Malvestiti and Dantas, 2018; C. Wang et al., 2019). The Fenton reaction, which is based on the use of a mixture of iron ions (Fe^2+^) and H_2_O_2_ that generates ^•^OH under mild acidic conditions, has been successfully tested as an attractive disinfection alternative (García-Fernández et al., 2019; Rodríguez-Chueca et al., 2012). Nevertheless, the large chemical consumption of H_2_O_2_ and the need to add and maintain appropriate ionic Fe concentrations in an acidic medium, coupled with the requirement to remove the iron species and neutralize the acid of the aqueous effluent after treatment, constitute major cost barriers for large-scale applications (Brillas and Martínez-Huitle, 2015; Fernández et al., 2018; García-Espinoza et al., 2019; Robles et al., 2020a, 2020b).

In the context of the cost limitation of AOPs, the electrochemical advanced oxidation processes (EAOPs) stand out as environmentally friendly approaches due to the possibility of generating reactive species using electric current. For this reason, they are in general low cost processes, which are easily operated and therefore constitute an efficient option for the inactivation of an extensive variety of pathogens, oscillating from virus, bacteria, parasites and algae (Lacasa et al., 2019; Martínez-Huitle et al., 2015; Martínez-Huitle and Brillas, 2008).

The effectiveness of the EAOPs such as electrochemical oxidation (Anfruns-Estrada et al., 2017; Bruguera-Casamada et al, 2016, 2017; Candia-Onfray et al., 2018; Cano et al., 2016; Corona-Bautista et al., 2021; Cotillas et al, 2018, 2020; Frontistis et al., 2017; Guitaya et al., 2015; Hernández-Pimentel et al., 2020; Herraiz-Carboné et al, 2020a, 2020b; Medrano-Rodríguez et al., 2020; Mousset et al., 2018; Pacheco-Álvarez et al., 2019), electrocoagulation (Anfruns-Estrada et al., 2017; Villalobos-Lara et al., 2021), electro-Fenton (Campos et al., 2020; Droguett et al., 2020; Olvera-Vargas et al., 2019; Ren et al., 2020; Robles et al., 2020a; Salazar et al., 2019; Thiam et al., 2020) and coupled EAOPs with other technologies (Cotillas et al., 2016b; Hashim et al., 2020; Jhones dos Santos et al., 2021; Llanos et al., 2015) has been proven by several research works worldwide on the inactivation of pathogens and the treatment of wastewater.

In the framework of EAOPs, the inactivation of pathogens can be achieved by direct oxidation which occurs when the pathogen reacts directly at the anode’s surface (Comninellis, 1994; Comninellis and Nerini, 1995; Panizza, 2010) or by quasi-direct oxidation by physi- or chemi-sorbed ^•^OH radicals in the anode’s surface vicinity (Comninellis et al., 2008; Groenen-Serrano, 2018; Groenen-Serrano et al., 2013; Marselli et al., 2003). Furthermore, indirect oxidation can also take place by means of the electrochemical generation of a mediator such as O_3_, H_2_O_2_, active chlorine or active bromine, among others, which in turn can disinfect aqueous effluents in the bulk solution (de Moura et al., 2015; García-Espinoza et al., 2018; Kanakaraju et al., 2018; Martinez-Huitle and Ferro, 2007; Martínez-Huitle and Panizza, 2018).

The combination of the electrochemical processes with other technologies in order to increase the efficiencies obtained by the single electrolytic processes is a popular research topic (Brillas, 2020; Martínez-Huitle et al., 2015). Particularly, the improvement of the EAOPs disinfection performance can be achieved by coupling an external source of energy such as UV or visible irradiation, giving rise to photo-assisted EAOPs in which electrogenerated mediators are photo-activated by irradiation, resulting in an increased generation of oxidizing agents in the anode’s surface as well as in the bulk of the aqueous solution (Cotillas et al., 2016a; de Vidales et al., 2015; Martínez-Huitle et al., 2015; Sirés et al., 2014; Souza et al., 2014).

**Fig. 1:**
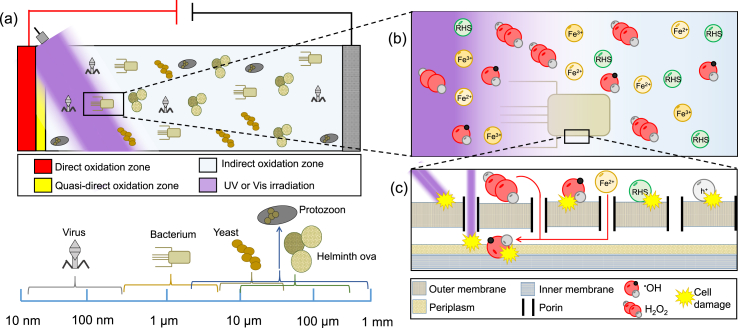
Inactivation of microorganisms in water by (a) photo-assisted EAOPs via direct, quasi-direct and indirect oxidation, (b) electrogenerated oxidants, photons and (c) cell damage of the outer membrane of a microorganism by h^+^, ^•^OH, RHS and photons.

Fig. 1 depicts a scheme of a photo-electrochemical reactor where the inactivation of microorganisms (from virus to parasites eggs (nm to μm of size)), can be achieved by the three inactivation mechanisms defined by the particular reaction zone in which disinfection occurs (Fig. 1a). As can be seen in Fig. 1b, the electrogenerated species such as reactive oxygen species (ROS), reactive halogen species (RHS) or photo-generated holes (h^+^) are capable of interacting with the microorganism eventually causing damage to the components of the membrane cell, attacking cellular components and leading to further oxidative damages. As can be observed in Fig. 1c, the bacterial envelope, composed of the outer membrane and the periplasm layer, have the largest probability of being exposed to ROS attack when compared to other cellular components (An et al., 2016; Sun et al., 2016). Fig. 1c also shows a scheme on which it can be observed that Fe^2+^ and H_2_O_2_ species could permeate through cell porins, thus generating the ^•^OH species which in turn, cause damage to the periplasm and inner membrane of the microorganism.

It is important to highlight however that in order to achieve inactivation of a pathogen it is necessary to carry on at least one of the inactivation mechanisms, and hence, the complete degradation or mineralization of the pathogens is not essential to accomplish an efficient inactivation process.

In this context, this work offers an overview of the application of photo-assisted EAOPs for disinfection of water. The fundamentals and characteristics for the direct, quasi-direct and indirect oxidation disinfection paths are highlighted in order to understand the role of the different oxidizing agents that participate in microorganism’s inactivation. Finally, the perspectives of the photo-assisted EAOPs for disinfection purposes are discussed.

## 2 Infectious agents commonly found in water and wastewater

Since wastewater contain numerous types of microorganisms that constitute a threat to human health as well as to ecological stability (Lam et al., 2020), the discussion of the different photo-assisted EAOPs disinfection methods starts with a brief description of the different types of pathogens that are found in contaminated waters.

**Fig. 2:**
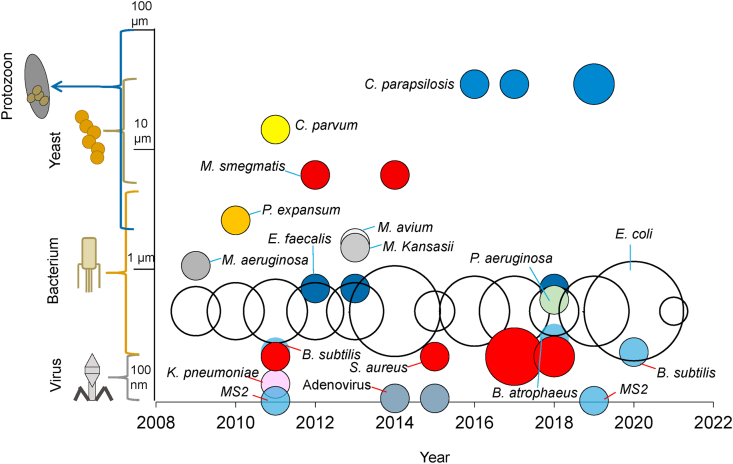
Proportion of scientific reports on the inactivation of pathogens by photo-assisted EAOPs.

As can be seen in Fig. 2, over the last 12 years there has been a large number of publications dealing with various pathogens whose size range between nm and μm. As it can be noted, the intensity of the circles is related with the amount of articles (n = 97) and the inactivation of *E. coli* by photo-assisted EAOPs shows the highest amount of literature reports (white circles, 68%), followed by *S. aureus* (red circles, 8.2%), *C. parapsilosis* (blue circles, 4.1%), and *E. faecalis* (dark blue circles, 3.1%); other pathogens are reported with less of 2% of the total.

Considering that the major groups of pathogenic organisms are: (a) bacteria, (b) viruses, (c) protozoans and (d) helminths (Sperling, 2008) it is important to point out that the initial microorganism density as well as the nature of mixed bacterial populations in water play an important role in the selection of a disinfection process, since the combination affects the type, cost and required treatment time for complete microbial inactivation (Venieri et al, 2012, 2013). In this regard for example, while bacteria are physically protected at high population density values, suspended solids and turbidity can decrease the performance of the photo-assisted EAOPs due to irregular light intensity scattering (Rather and Lo, 2020). Aquatic media for example is characterized by the presence of ions such as Cl^−^ (up to 755 mg/L), as reported by García-Espinoza and Mijaylova Nacheva, 2019; and Magazinovic et al., 2004 and Br^−^ (10 μg/L to 67 mg/L), as reported by Heeb et al., 2014, which may exert an important effect in the disinfection process. In the presence of NaCl or NaBr for instance, photo-electrocatalytic inactivation efficiencies of the pathogens are remarkably enhanced due to the formation of RHS (Nie et al., 2014a). The production of RHS can also be successfully couped to a high electrolyte concentration which results in fast inactivation due to high ionic conductivity values (Domínguez-Espíndola et al., 2019). In this regard, it is important to note that due to the complex combination of effects in real effluents, many studies employ synthetic solutions of known composition so that salts such as Na_2_SO_4_, NaNO_3_ and NaCl are used to achieve a convenient conductivity level for the electrochemical events to take place. In any case, real effluents are usually contaminated with a wide variety of pathogens and in this context, some of the major infectious agents reported in the literature that have been dealt with photo-assisted EAOPs, are characterized by different size, from virus to protozoan.

The adenoviruses for example, with average size of 0.08 μm, typically cause infections in respiratory tract, gastroenteritis and conjunctivitis (Goikhman et al., 2020); the MS-2 bacteriophage, on the other hand, with an average size of 0.03 μm, is an indicator for human enteric virus, which demonstrates notable resistance to photo-catalytic disinfection (Cho et al., 2011). Both adenoviruses and the bacteriophage represent 4.1% of the reports related with their inactivation by photo-assisted EAOPs (Fig. 2).

A pathogen with a higher size than viruses is *K. pneumoniae,* with an average size of 0.4 μm, which is a type of Gram-negative bacteria that can cause different types of infections, including pneumonia. Increasingly, *K. pneumoniae* bacteria have developed antimicrobial resistance, most recently to the class of antibiotics known as carbapanems (Srinivasan and Patel, 2008) and its inactivation by photo-assisted EAOPs has been scarcely studied with only 1% of the literature reports (Fig. 2).

*B. atrophaeus and B. subtilis* are bacillus, Gram-positive, aerobic and spore-forming, which are commercially available bacteria that can be used as a sterilization biological indicator (Sella et al., 2015). These bacilli show sizes from 1 to 1.5 μm and 3.1% of the articles of the photo-assisted EAOPs, report their inactivation (Fig. 2).

*S. aureus* on the other hand*,* is Gram-positive bacterium, a major bacterial human pathogen that causes clinical manifestations such as bacteremia, infective endocarditis, skin and soft tissue infections (Boucher and Corey, 2008). *S. aureus* presents similar size than bacillus, around 1 μm; and 8.2% of the articles surveyed, inform the performance of the photo-assisted EAOPs on its inactivation (see Fig. 2).

As it can be seen from data in Fig. 2, *E. coli* (2 μm of average size) is not only the most studied pathogen, with 68% of the articles, but also the main bacterium of the fecal (thermotolerant) coliform group, being present in large numbers in the feces of humans and animals. *E. coli* causes diarrhea by fecal oral transmission mechanism and therefore, it is the most researched out of all the pathogens not only because its presence indicates fecal contamination from human and animal origin but because its laboratory detection is relatively simple (Sperling, 2008).

*E. faecalis* on the other hand, is an important Gram-positive bacterium with an average size of 2.5 μm, which is frequent cause of many serious human infections, including urinary tract disease, endocarditis, bacteremia and wound infections. Diseases with *E. faecalis* can be especially troublesome because of their frequent resistance to multiple antibiotics (Kau et al., 2005). According to Figs. 2 and 3.1% of the articles related to photo-assisted EAOPs deal with the inactivation of *E. faecalis*.

*P. aeruginosa* on the other hand, is a rod-shaped Gram-negative bacterium, ubiquitous in soil and water as well as in animals and in plants with a contimous increase of its antibiotic resistance (Domínguez-Espíndola et al., 2019). This aerobic pathogen, with an average size of 2.3 μm, has been poorly evaluated with photo-assisted EAOPS with 1% of the literature reports.

*M. aeruginosa* is a photosynthetic cyanobacterium that plays an important role in global oxygenation; also, it forms water blooms in nutrient rich waters, causing water contamination and public health threats (Qu et al., 2018). According to Fig. 2, the average size of *M. aeruginosa* is 3 μm and only 1% of the articles of photo-assisted EAOPs report its inactivation.

The mycobacteria such as *M. avium, M. kansasii and M. smegmatis* have frequently been isolated from drinking water and hospital water distribution systems (Brugnera et al., 2013). The mycobacteria are potential pathogens involved in pulmonary or cutaneous diseases. There has been evidence that water can be the vehicle through which mycobacteria infect the human body; and chlorination is not efficient to fully inactivate them (Brugnera et al., 2012). The average sizes of the mycobacteria are 3–4 μm and 4.1% of the scientific articles report their inactivation.

With a high size of about 4.5 μm, *C. parvum* is a protozoan and a parasite that cannot survive without a host and commonly causes cryptosporidiosis, a diarrheal disease. *P. expansum* on the other hand, is a pathogen which causes the Blue mold disease, the most economically important postharvest disease of fruit and vegetables in storage (Errampalli, 2014). Both *C. parvum* and *P. expansum* have been scarcely assessed by means of photo-assisted EAOPs with 1% of the literature reports each one.

Finally, *C. parapsilosis* present an average size of 7 μm and it is the most frequently isolated agent in hospital settings and responsible for 80% of fungal infections contracted in these facilities. *C. parapsilosis* affects immunocompromised individuals including those who require prolonged use of intravenous catheter, such as dialysis and cancer patients (Pires et al., 2016; Souza et al, 2017, 2019). The inactivation of *C. parapsilosis* has been reported in 4.2% of the articles concerning photo-assisted EAOPs (Fig. 2).

As it can be noted, in the last years, photo-assisted EAOPs have been tested in the inactivation of several pathogens in aqueous solutions.

## 3 Photo-assisted disinfection of wastewaters using EAOPs

**Fig. 3:**
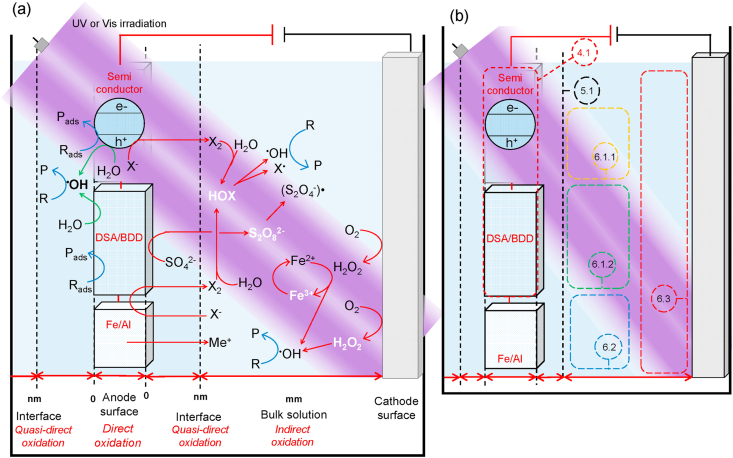
(a) Mechanisms and zones of reaction for direct, quasi-direct and indirect oxidization in photo-assisted EAOPs. Me^+^ = metal, X = Cl or Br, R_ads_ = microorganism, P_ads_ = inactivated microorganism. (b) section of this review in which each mechanism is discussed: 4.1 direct oxidation; 5.1 quasi-direct oxidation; 6.1.1 Indirect oxidation (inactivation) of pathogens using photo-anodes; 6.1.2 indirect oxidation (inactivation) of pathogens using DSA or BDD anodes; 6.2 photo-electrocoagulation; 6.3 cathodic processes.

The scheme shown in Fig. 3a summarizes the main reactions and mechanisms expected for the generation of oxidants by direct, quasi-direct and indirect oxidation processes that take place in photo-assisted EAOPs (photo-electrocatalysis, photo-electrochemical oxidation, photo-electrocoagulation and photo-electro Fenton processes). Fig. 3b on the other hand, depicts the section in this work in which each mechanism is discussed.

As can be seen in this scheme, there is a variety of oxidizing species that are produced in different zones of the reactor where they react with the pathogen membrane or most exposed structure thus producing inactivation. The inactivation process is therefore strongly dependent on the oxidation potential of the disinfection agent. Table 1 compiles the standard potential values of some oxidizing species that are generated in the photo-assisted EAOPs along with the oxidation mechanism as described by the scheme shown in Fig. 3a.

**Table 1:** Standard potential value of some oxidizing species and their oxidation mechanism.

Oxidation	Oxidizing species	Standard potential (V *vs.* SHE)
Direct	Direct electron transfer at anode surface	
	h^+^ on TiO_2_	3.20
Quasi-direct	^•^OH	2.80
Indirect	SO_4_^•-^	2.6
	O_3_	2.08
	^•^Cl	2.40
	S_2_O_8_^2-^	2.05
	^•^Cl_2_^_^	2.00
	Br^•^	1.96
	H_2_O_2_	1.76
	HClO pH 3-8	1.49
	Cl_2_ pH < 3	1.36
	HBrO	1.33
	O_2_	1.23
	Br_2_	1.09
	ClO^−^ pH > 8	0.89

**Fig. 4:**
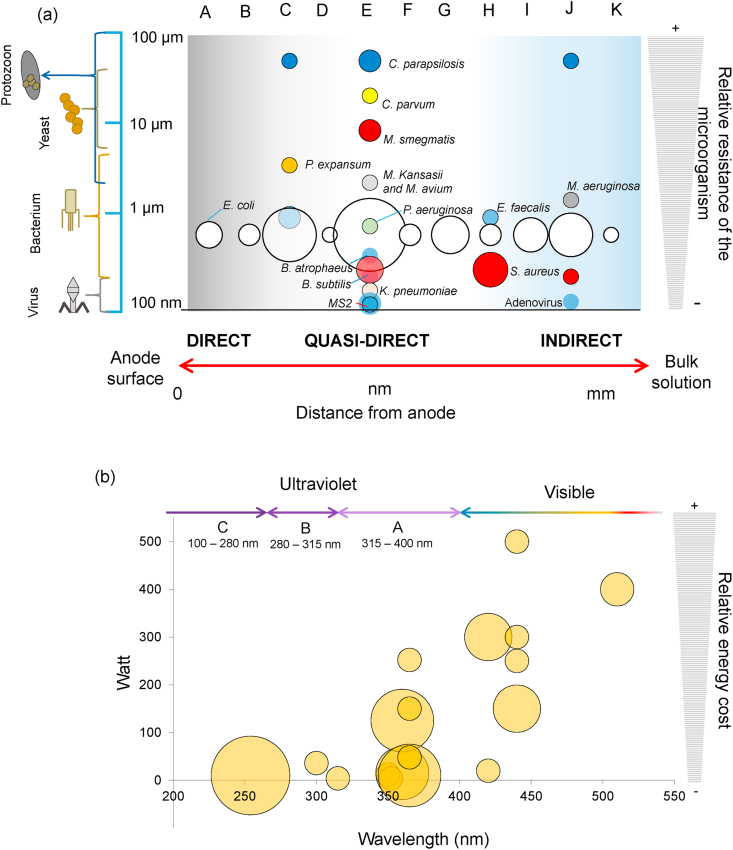
(a) Scheme relating the oxidation mechanisms of microorganism inactivation by photo-assisted EAOPs and the relative resistance of pathogens. A: Direct, B: Direct and indirect, C: Direct and Quasi-direct (h^+^ and ^•^OH), D: Direct, quasi-direct and indirect (h^+^, ^•^OH and RHS), E: Quasi-direct (^•^OH), F: Quasi and indirect (^•^OH and RHS), G: Indirect (electrocoagulation), H: Indirect (Fenton), I: Indirect (H_2_O_2_), J: Indirect (RHS), K: Indirect (sulphate radical). (b) Wavelength and power of the UV and Visible photon’s as a function of the relative number of papers (size of the corresponding circle) using photo-assisted EAOPs for disinfection purposes.

It is interesting to note that as expected, the most powerful oxidizing agents are those that react with the pathogen either on the surface of the electrode or very close to the electrode-solution interphase. In this regard, a classification of the oxidation mechanism reported on the literature (n = 95), can be made relating the distance from the anode’s surface with the type of microorganism. As it can be seen in Fig. 4a, most of the pathogen’s inactivation reports correspond to quasi-direct oxidation reaction with ^•^OH radical species (42.11%) reporting inactivation of pathogens ranging from small viruses to large protozoan microorganisms. The indirect oxidation mechanism reports, add up 33.7% of the reviewed articles, corresponding to an inactivation mechanism promoted by RHS (12.63%), followed by the Fenton reaction (8.42%), electrocoagulation (6.32%), H_2_O_2_ (5.26%) and sulphate radical (1.05%). The pure direct oxidation mechanism is scarcely reported (3.16%), nevertheless, more than one oxidation mechanism could be taking place: direct and indirect (2.11%), direct and quasi-direct (h^+^ and ^•^OH) (16.84%), direct, quasi-direct and indirect (h^+^, ^•^OH and RHS) (1.05%) and quasi and indirect (^•^OH and RHS) (1.05%) (see Fig. SM1-a). The size of the microorganisms that are inactivated in these processes can be related to their relative resistance to the attack of the photo-electrogenerated chemical oxidants.

For photo-assisted EAOPs a source of photons characterized by UV or Visible energy has been employed. Fig. 4b shows a classification of the emission wavelength of the photonic source and the corresponding power. As expected, the use of UV energy represents 72.54% of the published articles related to the disinfection of water by photo-assisted EAOPs (21.57% UVC, 3.92% UVB and 47.06% UVA). On the other hand, the cheaper and widely available use of visible energy was reported on the 27.46% of the articles (see Fig. SM1-b). The visible energy is typically supplied by solar simulators equipped with Xe lamps. Since UV photons are more energetic than visible light photons, more energetic h^+^ are generated under UV irradiation, which should produce faster oxidizing reactions and more efficient disinfection processes. As a consequence, the use of UV energy still represents a better alternative when compared to visible radiation for the promotion of reactive species. However, it is important to develop disinfection processes that, instead of UV energy, are capable of efficiently using visible radiation.

## 4 Direct oxidation

### 4.1 Fundamentals of direct oxidation

As it is shown in Fig. 3a, the direct oxidation or inactivation of the pathogens occurs right at the anode or photo-anode surface. These electrodes can be the so-called dimensionally stable anodes (DSA), mixed metal oxides (MMO) or boron doped diamond (BDD) electrodes. Using EAOPs, direct electron extraction after pathogen adsorption on the anode surface can result in effective microorganism inactivation without the involvement of other substances (Eq. (1)) (Martínez-Huitle and Panizza, 2018; Panizza and Cerisola, 2007, 2009).

(1) $${\text{R}}_{\text{ads}}-z{\text{e}}^{-}\to {\text{P}}_{\text{ads}}$$

The mechanism shown in Eq. (1) is commonly known as direct oxidation and it is characterized by the exclusive mediation of electrons, which in addition, must capable oxidizing the pathogen’s external structure at potentials lower than that of oxygen evolution (Garcia-Segura et al., 2018; Panizza and Cerisola, 2007). Since the electron transfer that produces inactivation is usually slow, the adsorption of the pathogen on the anode controls the process and does not lead to the complete pollutant mineralization (Garcia-Segura et al., 2018).

The photo-assisted electrocatalytical oxidation (also called photo-electrocatalysis (PEC)) has received increased attention due to the combined advantages of photo-catalytical and electrochemical processes (He et al., 2020). By using semiconductor materials such as TiO_2_, WO_3_ and ZnO (Garcia-Segura and Brillas, 2017; Li et al., 2011; Nie et al., 2014a) photo-anode illumination of a semiconductor–electrolyte interface with photons having enough energy to generate electron–hole (e^−^/h^+^) pairs (Daghrir et al, 2013, 2014) can promote effective separation of the photo-generated carriers within the space charge layer. In this way, photo-generated electrons and h^+^ are separated and pathogens are inactivated in the photo-anode providing at the same time electrons that are transported to cathode through an external circuit (Eq. (2)) (Lam et al, 2019, 2020; Philippidis et al., 2010).

(2) $$\text{Semicond}+hv\to \text{Semicond}-{\text{e}}_{\text{cb}}^{-}+\text{Semicond}-{\text{h}}_{\text{vb}}^{+}$$

In Eq. (2) the subscripts *cb* and *vb* denote the conduction and valence bands, respectively (Domínguez-Espíndola et al., 2019; Venieri et al., 2013).

In this context, a good example of photo-assisted direct oxidation of a representative pathogen agent was reported by Yu et al. (2008) who inactivated *E. coli* using photo-electro generated h^+^ at ZnIn_2_S_4_ photo-anodes (Yu et al., 2008). As it was shown in this direct photo-electrochemical process, h^+^ were found to be the major reactive species and therefore, the photo-catalytically generated h^+^ species act as a bactericide (Jin et al., 2020b; Li et al, 2011, 2013; Rather and Lo, 2020). This idea is consistent with the report of (Nie et al., 2014a) who found that photo-generated h^+^ species were predominantly responsible for PEC inactivation processes (Nie et al., 2014a).

For direct disinfection processes, a careful reactor arrangement is particularly important in order to effectively carry out the reaction between the photo-generated oxidant and the pathogen agent. A study by (Pablos et al., 2017a) for example, found that photo-generated h^+^ at the surface TiO_2_ electrodes covered by *E. coli,* showed significant differences in bacterial inactivation based on the way in which the reactor’s illumination set up was arranged. In his way, *E. coli* inactivation was noticeably diminished when the photo-electrodes were illuminated from the back side of the reactor, away from the region in which the surface adsorbed bacteria were located (Pablos et al., 2017a). The white circle at the left side of the Fig. 4a shows that the direct inactivation mechanism has been studied only with *E. coli.*

## 5 Quasi-direct oxidation

### 5.1 Fundamentals of quasi-direct oxidation

As it can be seen in the black dotted lines in Fig. 3a, the quasi-direct oxidation occurs few nm from the photo-anode or anode surfaces. In the case of oxidation of pathogens due to ^•^OH radical species produced by water oxidation at the anode surface, the inactivation process takes place in the vicinity of the electrode-solution interphase. In this way, a thin disinfection region aligned to the anode’s surface (<20 nm) is defined by the short lifetime as well as by the high chemical reactivity of the ^•^OH molecule. Therefore, as can be seen in Eq. (3), a quasi-direct oxidation process of pathogen agents take place in the vicinity of the anode surface where powerful oxidant species are generated from water or electrolyte electrooxidation reactions (Comninellis et al., 2008; Groenen-Serrano, 2018; Groenen-Serrano et al., 2013; Marselli et al., 2003).

Hence, depending on the applied potential, the inactivation of pathogens is known to occur through direct electron transfer in the potential region before oxygen evolution or by means of quasi-indirect oxidation via electrogenerated ^•^OH (Anglada et al., 2009; Panizza and Cerisola, 2009). Since direct and quasi-direct oxidation take place at or near the electrode surface, the concomitant reactions are nearly always mass transfer limited with a preponderant effect of the transport rates towards and from electrodes (Garcia-Segura et al., 2020).

In addition, the oxidation reaction of water for the generation of ^•^OH is always in competition with the secondary reaction of anodic dissociation of these radicals in oxygen and with the oxygen evolution reaction (see Eqs. (4), (5)) (Amadelli et al., 2000).

(3) $$\text{M}+{\text{H}}_{2}\text{O}\to {\text{M}}^{•}\left(\text{OH}\right)+{\text{H}}^{+}+{\text{e}}^{-}$$

(4) $$2{\left({\text{OH}}_{\text{ads}}\right)}^{•}\to {\text{O}}_{2}+2{\text{H}}^{+}+2{\text{e}}^{-}$$

(5) $$2{\text{H}}_{2}\text{O}\to 4{\text{H}}^{+}+{\text{O}}_{2}+{\text{e}}^{-}$$

While the ^•^OH radical species is considered as the main promoter for the inactivation of pathogens in quasi-direct disinfection reactions due to its high oxidation potential (Brillas, 2020; Martínez-Huitle and Brillas, 2009; Martinez-Huitle and Ferro, 2007) its activity is strongly related to its interaction with the anode’s surface (Comninellis et al., 2008). In this way, non-active anodes such as boron doped diamond (BDD) are preferred due to their high oxygen over-potential, achieving the oxidation of organic compounds by an electrochemical step mediated by physi-sorbed ^•^OH radicals. Active anode materials such as IrO_2_ or RuO_2_ on the other hand, exhibit lower oxygen over-potential and oxidation effectiveness; since the surface of these materials can be oxidized thus limiting the accumulation of ^•^OH (Polcaro et al., 2002).

Photo-assisted electrocatalytic processes have been largely explored in recent times for water disinfection due to their high efficiency, operability under ambient conditions and the associated low-cost of the equipment (Cho et al., 2019; Egerton, 2011; Garcia-Segura and Brillas, 2017; Rather and Lo, 2020). In these processes, the potential gradient forces the electrons towards the cathode, thus promoting the photo-generated h^+^ reaction at the anode with water to yield ^•^OH radicals (Eq. (6) – (8)) (Garcia-Segura and Brillas, 2017; Gupta et al., 2019; He et al., 2020; Venieri et al., 2013).

(6) $$\text{Semicond}-{\text{h}}_{\text{vb}}^{+}+{\text{H}}_{2}\text{O}\to \text{Semicond}-•\text{OH}+{\text{H}}^{+}$$

(7) $$\text{Semicond}-{\text{h}}_{\text{vb}}^{+}+{\text{OH}}^{-}\to \text{Semicond}-•\text{OH}$$

(8) $$\text{Semicond}-{\text{e}}_{\text{cb}}^{-}+\text{Semicond}-{\text{h}}_{\text{vb}}^{+}\to \text{recombination}$$

As it is shown in Fig. 4a, letter E, the quasi-direct oxidation is the most informed inactivation mechanism with 42.1% of the total reports of photo-assisted EAOPs for water disinfection, and it has been tested on several pathogens, paryicularly *E. coli* with 24.2%, followed by *S. aureus* with 3.1%, *C. parapsilosis*, MS2 coliphage and *M. smegmatis* with 2.1%.

### 5.2 Reports of quasi-direct oxidation

#### 5.2.1 TiO_2_

In this context, TiO_2_ is by far the most studied photo-catalyst for quasi-direct oxidation due to its efficient photo-activity, high chemical stability and non-toxicity (Daghrir et al., 2013; Fujishima and Zhang, 2006; He et al., 2020). For example, a TiO_2_ photo-anode was integrated in a portable water disinfection device by Montenegro-Ayo et al. (2020), in which a 365 nm light emitting submergible two-sided diode lamp is powered by a rechargeable battery. This disinfection device was able to achieve 5-log inactivation of *E. coli* in 10 s of treatment in model water samples by means of photo-electrogenerated ^•^OH radicals (Montenegro-Ayo et al., 2020).

Photo-catalytic inactivation of *E. coli* and *E. faecalis* in water samples can also be enhanced by applying a positive potential on TiO_2_/Ti films under simulated solar radiation (Venieri et al, 2012, 2013). The quantity of *E. coli* cells were reduced by approximately 6 orders of magnitude after 15 min of PEC treatment in water at 2 V of applied potential and at an initial concentration of 10^7^ CFU mL^−1^ (Venieri et al., 2013). A 6.2 log reduction in *E. faecalis* population was also achieved after 15 min of PEC treatment in water at 10 V of applied potential and an initial concentration of 10^7^ CFU mL^−1^ (Venieri et al., 2012).

Antibiotic-resistance bacteria and antibiotic-resistance genes such as *E. coli* S1-23 and bla_TEM-1_ and aac(3)-II were chosen by Jiang et al. (2017) as pathogens to prove the effectiveness of a photo-electrocatalytic process for their inactivation using TiO_2_ nanotubes (NTN). Their results showed an effective inactivation and an intracellular and extracellular leakage of the bacteria towards the environment (Jiang et al., 2017).

Additional efforts have been made in order to enhance the photocatalytic properties of TiO_2_. In this regard, the enrichment of Ti^3+^ species within the semiconductor structure (self-doping) by cathodic polarization of TiO_2_ improves its electronic and optical properties (Cho et al., 2019). In this way, oxygen vacancies (e.g. Ti^3+^ self-doping), are formed within the lattice of TiO_2_ NTN arrays during the electrochemical reduction process of pristine TiO_2_ at different negative potentials ranging from −1.2 to −1.5 V. Disinfection with the TiO_2_ NTN arrays were observed to enhance the photo-electrocatalytic activity in the UV and visible regions of the electromagnetic spectrum (Liao et al., 2014) yielding a photo-current density 250% higher than that of pristine TiO_2_. Highly ordered TiO_2_ NTN arrays directly synthesized by anodizing Ti foil have also attracted considerable attention due to their unique chemical and physical properties as well as their excellent capability for instant inactivation and rapid decomposition of *E. coli*. (Li et al., 2016; Liu et al., 2013; Nie et al., 2014a; Sun et al., 2014). For instance, photo-electrocatalytic disinfection using a highly ordered TiO_2_ TNT array, resulted in a 100% inactivation of *E. coli* (1.0 × 10^7^ CFU mL^−1^) within 97 s, which was almost 2.2 times faster than that using a nanoparticulated TiO_2_ photo-anode (Liu et al., 2013).

The high reactivity of {001} facets exposed in nano-sized single crystals and superior electron transport properties of the TiO_2_ NTN array, enhance the *E. coli* removal efficiency of TiO_2_-based photo-catalysts (Li et al., 2016). In addition to the crystalline phase anatase, pure rutile TiO_2_ photo-anodes with 100% exposed {111} facets possess visible light activity (Liu et al., 2014). The origin of the visible light activity of such {111} faceted rutile TiO_2_ can be associated to Ti^3+^ doping achieving 99.97% inactivation of 1.0 × 10^7^ CFU mL^−1^ *E. coli* cells within 10 min of photo-electrocatalysis treatment (Liu et al., 2014). Ribeiro et al. (2015) also compared the performance of photo-electrocatalytic and photo-catalytic processes using TiO_2_; finding that the photo-electrochemical treatment was more efficient than the photo-catalytic one for *S. aureus* inactivation (Ribeiro et al., 2015).

For Gram-negative bacteria, such as *E. coli*, the outer membrane, the cell wall and the cytoplasmic membrane are located in the outer part of the cell, and thus, these parts of the microorganism are the most suitable targets for ^•^OH radical attack (An et al., 2016). In this regard, An et al. (2016) found that the use of a TiO_2_ NTN array photo-anode causes oxidative damage to the protein membrane, particularly to bacterial energy metabolism such as respiration and adenosine triphosphate generation, which results in a lethal effect (An et al., 2016).

As opposed to the effective quasi-direct oxidation produced by ^•^OH radical species, a report by (Zhang et al., 2020b) showed that direct h^+^ transfer exert little effect on bacteria inactivation, probably due to h^+^ scavenging phenomena caused by the NaCl electrolyte. The concomitant transfer of photo-generated electrons to the cathode on the other hand, results in the production of ROS; such as H_2_O_2_ and ^•^O_2_^−^, which were found to play a critical role in the inactivation of *E. coli* bacteria (Guan et al., 2020).

Other TiO_2_ NTN arrangements have been explored for disinfection purposes. A Ag/AgBr/TiO_2_ NTN array electrode , was prepared and used to study the oxidative attack of photo-electrocatalytically produced ^•^OH, O_2_^•−^, h^+^ and Br^0^ to the external and internal membrane of *E. coli.* (Hou et al., 2012). Furthermore, in photo-electrocatalytic processes, electronegative *E. coli* membrane surfaces can get in direct contact with photo-generated h^+^ and ^•^OH radical species due to the strong adsorption promoted by the positive polarization potential (electromigration) of the anode surface (Cho et al, 2011, 2019; Kang et al., 2010; Pablos et al, 2017a, 2017b).

#### 5.2.2 Doped photo-anodes

As it was previously mentioned, TiO_2_ is by far the most popular semiconductor material for photo-electrocatalytic applications. One major disadvantage of this material however is related to its high energy band gap which limits its light absorption properties to the UV region of the electromagnetic spectrum. In this context, and in order to open the possibility for solar energy (400–780 nm) usage, modified TiO_2_ semiconductor photo-anodes with band−gap energies between 1.7 and 2.0 eV are ideal candidates (Zhang et al., 2020b). In this way, the absorption spectra of TiO_2_ can be extended well into the visible region by doping the semiconductor lattice with anions such as nitrogen. Therefore, an N-doped Ti/TiO_2_ photo-anode was prepared, characterized and tested for the removal of fecal coliform from a municipal wastewater facility under visible energy irradiation; reaching log-inactivation values higher than 1.2 units (Daghrir et al., 2014). The mixing of *2p* states in the valence band of N and O, results in narrowing of the TiO_2_ semiconductor band-gap and the consequent shifting absorption onset to lower energies.

N-doped TiO_2_ NTN as photo-anode substrates were also evaluated for the inactivation of *E. coli* by (Pablos et al., 2017b) and in contrast to the effect observed by Daghrir et al. (2014), these photo-anode structures showed that while electrochemically assisted photo-catalytic inactivation of bacteria occurred under UV–Vis irradiation, no effect was observed under visible irradiation (Daghrir et al., 2014; Pablos et al., 2017b).

Another example of this type of materials was reported by He et al. (2020) who prepared Ti plates with highly orientated anatase on which TiO_2_ NTN arrays decorated by antimony doped tin oxide (SnO_2_–Sb) and silver nanoparticles (Ag) were constructed. In this novel photo-anode the SnO_2_–Sb/Ti promotes the generation of ROS and the Ag content on TiO_2_ substantially boosted the activity of the catalyst by increasing the separation of photo-induced e^−^/h^+^ pairs (He et al., 2020; Liu et al., 2014). This feature allowed a high *E. coli* inactivation performance as compared with a substrate in which the incorporation of SnO_2_–Sb or Ag were absent.

Domínguez-Espíndola et al. (2019) on the other hand, reported a fast and total inactivation of *P. aeruginosa* using Ag-decorated TiO_2_ photo-anodes deposited on indium tin oxide. The set up also consisted on a stainless-steel (SS) cathode and UVA irradiation. Their experiments revealed total inactivation of the pathogen within 5 min using coatings loaded with 4% w/w of Ag, 0.25 M Na_2_SO_4_ as electrolyte and 1.70 V *vs.* Ag|AgCl (3 M KCl) as the applied bias potential (Domínguez-Espíndola et al., 2019). As expected, the presence of Ag was identified to significantly increase the response of TiO_2_ towards bacterial inactivation. Upon an applied cell potential of 1.5 V the TiO_2_/Ag (4% w/w) photo-anode also achieved complete inactivation of fecal coliform bacteria in the solution within 6 min (Domínguez-Espíndola et al., 2017). Brugnera et al. (2014) also prepared a Ti/TiO_2_–Ag photo-anode that was used in photo-electrocatalytic disinfection experiments, achieving full inactivation of *M. smegmatis* in 3 min using UV irradiation and 99.6% in 30 min employing visible irradiation (Brugnera et al., 2014). A previous study reported one year before by the same group, pointed out that a total mycobacterium inactivation with an initial population of 7.5 × 10^4^ CFU mL^−1^, was completely elicited within 3 min of treatment using a Ti/TiO_2_–Ag photo-anode. The presence of Ag nanoparticles in the electrode were observed to be responsible for a 1.5 times larger degradation rate constant when compared to an unmodified Ti/TiO_2_ anode (Brugnera et al., 2013).

TiO_2_ NTN array electrodes have also been coated with Ag nanoparticles (16% w/w), showing excellent performance for the disinfection of water containing *M. smegmatis*. In this way, 100% inactivation was achieved after 3 min of photo-electrocatalytic treatment (5.1 × 10^3^ CFU mL^−1^ in 0.05 M Na_2_SO_4_, pH 6, applied potential of 1.5 V and UV irradiation) (Brugnera et al., 2012).

Ag has therefore been proven to induce effective antibacterial activity, increasing the visible light excitation of TiO_2_, fostering charge transfer events at the solution/semiconductor interphase, facilitating the production of ^•^OH radicals and promoting as a consequence the fast and effective inactivation of microorganisms (Brugnera et al., 2014; Domínguez-Espíndola et al, 2017, 2019; He et al., 2003; Rahmawati et al., 2011). Ag also alters the transport system within the cell membrane resulting in catastrophic permeability, osmoregulation, electron transport and respiration events that eventually lead to cell death (Mafa et al., 2020). Domínguez-Espíndola et al. (2017) and Domínguez-Espíndola et al. (2019) also reported that Ag acts as photo-generated electron-trapping sites that prevent the recombination of photo-generated e^−^/h^+^ pairs; thus increasing the bacterial inactivation rate (Domínguez-Espíndola et al, 2017, 2019). However, according to Pires et al. (2016), the incorporation of Ag into Ti/TiO_2_ electrodes does not improve the inactivation of *C. parapsilosis* as expected; suggesting that Ag nanoparticles may act in some cases as recombination sites for e^−^/h^+^ pairs (Pires et al., 2016).

#### 5.2.3 ZnO

In addition to TiO_2_, some other wide band gap semiconductor materials have been explored for water disinfection processes. In this regard, Zinc oxide (ZnO) has attracted much attention since it is a widely available n-type semiconductor material (band gap>3 eV), that is characterized by its low cost, non-toxicity and high photo-activity (Lam et al., 2020; Lee et al., 2017; Sapkal et al., 2012). Lam et al. (2020) for example, developed a photo-catalytic fuel cell based on ZnO/Zn or TiO_2_/ZnO/Zn anodes in which electricity production was coupled with bacteria disinfection. The corresponding experiments reached complete *E. coli* inactivation after 60 min of treatment by the combined effects of h^+^ injection and ^•^OH production (Lam et al., 2020). Gupta et al. (2019) on the other hand, reported the disinfection performance of a ZnO/CuI nanorod array that was grown on a fluorine-doped tin oxide (FTO) substrate. The novel arrangement of materials in the photo-anode structure produced a potential barrier that was assumed to prevent charge carrier recombination which resulted not only in outstanding charge separation and extended visible light absorption, but also in an excellent activity towards bacterial inactivation (Gupta et al., 2019). ^•^OH, h^+^ and superoxide radicals were identified as the species responsible for inactivation of *E. coli* by means of an oxidative stress mechanism that lead to membrane damage*;* achieving 95% inactivation when compared to UV illuminated samples that reached 20% (Sapkal et al., 2012).

#### 5.2.4 WO_3_

Tungsten trioxide (WO_3_) is another attractive semiconductor material that has been widely explored due to its relatively low cost and ability to absorb visible light (band gap of 2.5–2.8 eV). This feature extends the photo-catalytic activity of the semiconductor anode into the visible light region leading to utilization of approximately 30% of solar radiation, as opposed to pure TiO_2_ (Juodkazytė et al., 2020; Koo et al., 2019; Scott-Emuakpor et al., 2012; Souza et al., 2017). In this context, Souza et al. (2017) compared the inactivation of C. *parapsilosis* using Na_2_SO_4_, NaNO_3_ and NaCl as electrolytes in photo-electrocatalytic process employing a W/WO_3_ photo-anode (Souza et al., 2017). The inactivation mechanism of the microorganism was found to be associated with the attack of ^•^OH radicals to the cell membrane, where the microorganism-catalyst contact takes place. Souza et al. (2019) reported that the ^•^OH radical, responsible for microorganism death, were readily produced on the electrode surface and seemed to be the main reactive species, even in a high chloride ion concentration solution (Souza et al., 2019).

#### 5.2.5 Cu_2_O, CuO

Cooper oxide is another semiconductor material that has been investigated as photo-electrode material due to its abundance, low cost, non-toxicity, inherently p-type character, high optical absorption and good charge transport properties. Cu_2_O is a direct-gap semiconductor with a relative low band gap energy of ∼2.1 eV (Lam et al., 2020; Masudy-Panah et al., 2016).

In this way, CuO catalyst particles have been used in a stirred photo-reactor using FTO and Pt as anode and cathode, respectively for *E. coli* inactivation. The experiments showed an increase of more than three times in the photo-electrocatalytic inactivation rate process compared with the one obtained in the photo-catalytic experiment performed in the absence of an external potential bias (Eswar et al, 2018a, 2018b).

#### 5.2.6 Photo-anodes prepared or mixed with activated carbon or graphite

Another interesting approach for the development of photo-active semiconductor anodes consist on the addition of carbonaceous material to the semiconductor substrate. Mesones et al. (2020) for example, reported a photo-electrochemical three-dimensional reactor using a commercial anode of RuOx/Ti and an illuminated photo-catalyst of granular activated carbon (GAC)-TiO_2_ composite that was designed to work as a bipolar electrode (Mesones et al., 2020). Although several phenomena were assumed to take place in this system (adsorption, photolysis, electrolysis and photo-catalysis), bacterial inactivation was essentially mediated by ^•^OH radicals. In this way, a high value for the *E. coli* inactivation kinetic constant was obtained by combining photo-catalytic and electrochemical processes to produce ^•^OH radicals using a GAC-TiO_2_ composite, UVA radiation and 10 mA/cm^2^ of electric current density (Mesones et al., 2020). Furthermore, since the carbonaceous materials are usually characterized by a large number of surface active sites, excellent adsorption properties towards various organics and microorganisms result in a potential enhancement of the photo-catalytic activity of the composites of TiO_2_-carbonauceous materials (Nie et al., 2014b; Rahmawati et al, 2010, 2011; Zhang et al., 2011).

#### 5.2.7 Other photo-anode materials

Among photo-anodes, mediator-based Z-schemes between two different semiconductors have been reported to possess a higher charge separation efficiency due to e^−^/h^+^ annihilation by the mediator. For example, Rather and Lo (2020) reported a study of the performance of the g-C_3_N_4_/Ag/AgCl/BiVO_4_ heterojunction in the disinfection of *E. coli* present in sewage; achieving bacterium concentration values lower than those for permissible discharge limits (Rather and Lo, 2020). Spherical silver nanoparticles grown on silicon carbide (Ag@SiC) on the other hand, were evaluated for the photo-electrocatalytic inactivation of *E. coli* using Pt and FTO as counter and working electrodes, respectively, with an optimum Ag load of 3% w/w. The experiments allowed to find that at 3.0 V of applied potential, the rate of the photo-electrocatalytic process was four times higher than photo-catalytic oxidation (Adhikari et al., 2018). Coupling TiO_2_ with narrow-band gap semiconductor materials and metal composites has also been observed to be effective in enhancing the visible radiation activity and in simultaneous improvement of the charge separation efficiency. In this way, *E. coli* bacteria treated with a Ag/AgBr/TiO_2_ electrode were found to be substantially damaged, forming pits and holes in the cell walls (Hou et al., 2012). DSA anodes coupled with a Ti/TiO_2_ cathode can also be employed for the inactivation of adenovirus, with UV irradiation and 5 A of applied current, promoting the UV photo-assisted production of ^•^OH radical species (Monteiro et al., 2015).

## 6 Indirect oxidation

### 6.1 Fundamentals of indirect oxidation by photo-electrocatalytic production of reactive oxygen species (ROS) and photo-assisted-electro generation of active chlorine, active bromide and persulphate

The indirect oxidation or inactivation of pathogens is achieved through the electrochemical generation of a mediator in bulk solution such as reactive halogen species (RHS), persulphate (S_2_O_8_^2−^) or reactive oxygen species (ROS). The indirect oxidation takes place in the bulk solution (Fig. 3) where the electrogenerated species migrates few mm from the electrodes surface to interact with the microorganisms achieving their inactivation. In spite of to their convenient high oxidation power, ROS such as ^•^OH, O^•-^_2_, HOO^•^, O_3_ and H_2_O_2_ are characterized by important limitations such as a short lifetime and rapid recombination with several scavenger species (Li et al., 2011). In this context, other oxidants are the preferred choice and in this regard, it is well known that halogen radical compounds are effective bactericides which can be produced by photo-electrocatalytic oxidation of halide ions at the surface of illuminated photo-anodes (Li et al., 2013; Selcuk et al., 2006). This process is possible because these radicals are capable of forming stable di-halide radical anions (X^•-^_2_) in the presence of X^−^ (Li et al., 2011).

The photo-electrochemical generation of RHS (e.g. Cl_2_, HClO, ClO, Br_2_, HBrO and BrO), is therefore a process of high interest because it offers the possibility to produce the disinfectant chemicals on-demand and on-site (Juodkazytė et al., 2020). Active chlorine for example (Cl_2_, HClO, ClO^−^) can be generated by two main processes in the presence of a semiconductor material and UV irradiation. First, ^•^OH can be anodically produced by means of the oxidation of adsorbed water on the electrode surface (Eqs. (5), (6), (7)) and the products resulting from this reaction could in turn oxidize chloride ions (Eq. (9)). Furthermore, adsorbed chloride ions on the semiconductor surface could also be directly oxidized by photo-generated h^+^ under UV irradiation leading to the formation of Cl^•^/HOCl^•^ species (Eq. (10)). In the absence of competitive reactions with pathogens, the formation of active chlorine in solution takes place (Eq. (11)) (Fraga et al., 2009; Koo et al., 2019).

(9) $$\text{Semicond}-•\text{OH}+{\text{Cl}}^{-}\to {\text{HClO}}^{•}$$

(10) $$\text{Semicond}-{\text{h}}_{\text{vb}}^{+}+{\text{Cl}}^{-}\to \text{Semicond}-{\text{Cl}}^{•}$$

(11) $${\text{Cl}}^{•}+{\text{Cl}}^{•}\to {\text{Cl}}_{2}$$

As shown by Eq. (12), chlorine can also be produced by direct oxidation of dissolved Cl^−^ ions at the anode’s surface:

(12) 2Cl^-^ → Cl_2_ (aq) + 2e^−^

In any case, Cl_2_ in aqueous medium is hydrolyzed to produce hypochlorous acid which, depending on the pH of the solution, partially reacts with the solvent to give rise to hypochlorite ions (Eq. (13) – (14)).

(13) Cl_2_ (aq) + H_2_O ↔ HClO + Cl^−^ + H^+^

(14) HClO ↔ ClO^−^ + H^+^ pKa = 7.55

The subsequent photo-activation of active chlorine by the homolytic rupture of HClO is described by Eq. (15) (García-Espinoza et al., 2020; Sánchez-Montes et al., 2020).

(15) $$\text{HClO}+hv↔•\text{OH}+{\text{Cl}}^{•}$$

The standard potential (E° *vs.* SHE) of these chloro-species correspond to 1.36,1.49 and 0.89 V for Cl_2_, HClO and ClO^−^, respectively. As expected, the predominant form of the oxidant depends on the pH of the solution. In this way, while Cl_2_ is quite stable under strong acidic conditions (pH < 3), HClO and ClO^−^ are the predominant forms in the pH range of 3–8 and above 8, respectively (Sirés and Brillas, 2012). In addition to Cl_2_, HClO and ClO^−^, radicals such as ^•^Cl (E° = 2.4 V *vs.* SHE) react with Cl^−^ ions to produce long-lived and reactive Cl_2_^•−^ radicals (E° = 2.0 V *vs.* SHE) (Nie et al., 2014a; Wang et al., 2016).

As in the case of chlorine, bromide ions can be oxidized at the anode’s surface to produce hypobromous acid (Eq. (16)) which in turn, undergoes an acid/base equilibrium characterized by a pKa = 8.65 which, as can be seen in Eq. (14), is slightly smaller than that of hypochlorous acid (see Eq. (17)).

(16) Br^−^ + H_2_O ↔ HOBr + H^+^ + 2e^−^

(17) $$\text{HOBr}↔{\text{H}}^{+}+{\text{BrO}}^{-}$$

As shown by Eq. (18) – (20), Br^−^ ions can react at the anode’s surface either by direct oxidation (Eq. (18)) or by sequential h^+^ injection in photo-assisted processes producing intermediate Br^•^ radicals (Eq. (19) and (20)) (Selcuk et al., 2006).

(18) $$2{\text{Br}}^{-}\to {\text{Br}}_{2}+2{\text{e}}^{-}$$

(19) $${\text{Br}}^{-}\to {\text{Br}}^{•}+{\text{e}}^{-}$$

(20) $$2{\text{Br}}^{•}\to {\text{Br}}_{2}$$

As in the case of chloride, the resulting Br_2_ spontaneously hydrolyzes in aqueous medium leading to the formation of active bromide species (Br_2_, HOBr, BrO^−^). In the same way, the photolysis of active bromine results in the formation of ^•^OH and Br^•^ radical species (see Eq. (21)) (García-Espinoza et al., 2020; Selcuk et al., 2006).

(21) $$\text{HBrO}+hv↔•\text{OH}+{\text{Br}}^{•}$$

The reported standard potentials (E° *vs.* SHE) for the Br_2_/Br^−^, HOBr/Br^−^ and Br^•^/Br^−^ couples are 1.087, 1.33 and 1.96 V, respectively (Li et al., 2011). Since the pH of wastewater is usually close to neutrality, HBrO or HClO are typically the most stable forms of active bromine and chlorine. HClO and HBrO in turn, are characterized by E° *vs.* SHE values of 1.33 and1.49 V , which also explains the better disinfection performance of active chlorine when compared to active bromine.

It is also interesting to note that although the E° values of HOCl and HOBr are less oxidizing when compared to that of the ^•^OH radical species, they are in general more selective and can therefore react faster with electron-rich moieties such as the pathogen’s membrane (Wang et al., 2016). The RHS therefore show different reaction pathways to those exhibited by ^•^OH. RHS for instance, react preferentially via one-electron oxidation, dehydrogenation and addition to unsaturated C–C bonds, whereas ^•^OH reacts almost exclusively following the last two pathways (Grebel et al., 2010).

Sulphate ions on the other hand, are also another important kind of indirect oxidation agent in disinfection processes. In solution, sulphate anions interact with the anode, where persulphate ions and peroxydisulfuric acid are produced according to Eq. (22). In presence of UV radiation, the persulphate ions undergo photo-conversion to produce the sulphate radical as has been reported by Sirés and co-workers (Eq. (23)) (Sirés et al., 2014).

(22) 2SO_4_^2−^ ↔ S_2_O_8_^2−^ + 2e^−^

(23) $${\text{S}}_{2}{\text{O}}_{8}^{2-}+hv↔2{\left({\text{SO}}_{4}^{-}\right)}^{•}$$

In the last decade, (SO_4_^•-^) based treatment processes have gained attention due not only to the high oxidation power of sulphate radicals (E° = 2.5–3.1 V *vs.* SHE), but also to their nonselective reactivity (Zhi et al., 2020). The SO_4_^•-^ radical species is therefore a powerful oxidant that rapidly attacks a variety of organic compounds, as well as pathogen membrane structures; following reaction pathways that are similar to those that characterize ^•^OH chemistry, (Tan et al., 2021).

#### 6.1.1 Indirect oxidation (inactivation) of pathogens using photo-anodes

As it is the case for most direct photocatalytic studies, indirect processes have been widely explored using TiO_2_ semiconductor anode materials. Indirect photo-assisted inactivation of *E. coli* using a highly oriented TiO_2_ TNT anode was for example studied by Sun et al. (2016). These authors found not only high pathogen inactivation efficiencies but also that the ROS concentration in the PEC system was closer to the H_2_O_2_ concentration than to that of the ^•^OH radical species (Sun et al., 2016).

In a more sophisticated approach, an indirect photo-electrocatalytic process assisted by O_3_, using a TiO_2_ NTN photo-anode and DSA cathode, was found to completely inactivate *C. parapsilosis* in swimming pool water within 45 min of treatment. The study carried out by Kim et al. (2019) found that the efficient inactivation process was based on the generation of ROS which were provided by the photo-electrocatalytic oxidation of water, O_3_ production by photo-generated electrons at the TiO_2_ surface and the O_3_ reaction with water and UV photons (Kim et al., 2019).

MoS_2_ has a more suitable band gap energy (∼1.6–1.9 eV) when compared to other metal-oxide based semiconductors (Zhang et al., 2020b). The MoS_2_/MoO_x_ heterojunction for instance, allows to obtain complete inactivation of a 10^6^ CFU mL^−1^ *E. coli* solution prepared using a NaCl electrolyte and applying 0.5 V and visible irradiation. After 2 h of treatment, indirect complete pathogen oxidation was achieved by means of the photo-electrochemically produced H_2_O_2_ and ^•^O_2_^−^ which were found the key reactive oxidative species for *E. coli* inactivation (Zhang et al., 2020b). In a related study, Xiong et al. (2015) reported the complete inactivation of 7 log of *E. coli* within 2 h using an n-type Cu_2_O film electrode. In this case, H_2_O_2_, injection of photo-generated h^+^ and the inherent toxicity of the Cu_2_O film were found to be the main factors for pathogen’s inactivation (Xiong et al., 2015).

In addition of the use of ROS, indirect oxidation of pathogens using RHS is also very effective. (Nie et al., 2014a) for example, reported the use of a TiO_2_ NTN as photo-anode and UV irradiation at 365 nm, for the inactivation of *E. coli* in aqueous solution containing a bromide based electrolyte. These authors observed that by increasing the NaBr concentration in solution from 0.1 to 1 mM, the time required to achieve complete inactivation of *E. coli* sharply decreased 600 times due to the photo-electro-catalytical bactericidal performance of RHS (Nie et al., 2014a).

W/WO_3_ electrodes have also been successfully used for the photo-electrocatalytic inactivation of *C. parapsilosis* in wastewater containing a high concentration of Cl^−^ ions. Souza et al. (2019) for example reported inactivation of this pathogen within 1 min and almost 40% degradation of the by-products after 120 min of electrolytic treatment (Souza et al., 2019). Chlorine radicals (Cl^•^, Cl_2_^−•^) formed on the surface of WO_3_ photo-anode, have also been shown to have a high bacteria-killing power from experiments that assessed the inactivation of *B. subtilis* and *E. coli* in a NaCl electrolyte solution (Juodkazytė et al., 2020; Koo et al., 2019).

The important role of the reactive chlorine species in visible light-irradiated PEC experiments was identified by Koo et al. (2019) by comparing inactivation pathogen data in experiments carried out in the presence and in the absence of chlorine (Koo et al., 2019). The relative weight of ^•^OH and RHS in the PEC generation experiments reported in their work, revealed that the most important oxidant in the pathogen inactivation experiments corresponds to RHS since in the presence of chlorine ions, ^•^OH radical species are readily scavenged by Cl^−^ anions, which in turn results in the production of active chlorine. (Koo et al., 2019).

The PEC treatment of virus contaminated solutions in the presence of 1.0 mM Br, shows a high virucidal efficiency, enabling complete inactivation of a ∼1000 median tissue culture infectious dose of adenovirus population within 31.7 s. The highly efficient virucidal performance of PEC-Br treatments has been attributed to the high production of ROS and additional halogen viricide chemicals such as active bromide resulting from the PEC indirect-halide oxidation, as well as the direct inactivation at the photo-anode surface (Li et al., 2014).

#### 6.1.2 Indirect oxidation (inactivation) of pathogens using DSA or BDD anodes

When electrochemical oxidation processes and UV light are not considered to be simultaneously operating at the electrode-solution interphase, and rather complementing each other’s effect in a synergistic way, indirect disinfection processes using highly electro-catalytic and non-photoactive electrodes have been shown to be effective approaches. Under this perspective, it must be noted that UV irradiation represents a highly effective disinfection method that, although costly, can be combined with other disinfection procedures such as electrochemical oxidation (Cotillas et al., 2016a; Haaken et al., 2013; Martín de Vidales et al., 2016; Singla et al., 2020). The electrodes employed in this type of processes are usually the DSA, MMO or BDD electrodes (Panizza and Cerisola, 2005, 2009). The electrode material is a decisive criterion for a moderate energy consumption of UV coupled to an electrochemical oxidation reactor. For instance, a UV-electrochemical oxidation reactor operating with MMO electrodes requires 2–6 times lower electric charge input and shows a 5–10 times lower energy consumption compared to the average consumption of BDD electrodes (Haaken et al., 2013) and in contrast to BDD electrodes, no chlorite, chlorate and perchlorate were detected on processes using MMO electrodes. The unwanted reactivation of reversibly UV damaged *E. coli*, for example, is completely prevented at a concentration of electrogenerated total oxidants of 0.4–0.5 mg/L (Haaken et al, 2013, 2014). Furthermore, the problem of biofilm covering UV lamps in treating real wastewater photo-reactors, can be inhibited by means of the electrochemical generation of oxidants (Haaken et al., 2014). In *E. coli* contaminated synthetic urine for example, a MMO anode coupled with UV irradiation constitutes a good option for pathogen inactivation (complete removal after 30 min of treatment *vs.* 45 min using single electrochemical oxidation) in which full disinfection of wastewater depended not only on the production of disinfectant species but also on the concentration of chlorides added and on the concentration of reduced nitrogen species in solution (Singla et al., 2020). Under specific electrolyte composition conditions, MMO anodes coupled to UV irradiation also promotes the indirect oxidation of the pathogens by means of the activation of sulfite to generate persulphate. In these processes, the complete inactivation of 5.4-log of *E. coli* is achieved after 30 min of treatment (Chen et al., 2021).

It is important to point out that UV irradiation contributes to minimize the concentration of available hypochlorite in the electrochemical reactor and that it also has a positive effect on the prevention of the formation of undesirable and hazardous chlorate and perchlorate by-products (Cotillas et al., 2016a). In this regard, commercial reactors such as DiaCell® cell, equipped with a BDD anode, SS cathode and a UV irradiation source, has been successfully used for the disinfection of an *E. coli* contaminated real secondary effluent. This study revealed an improvement of four times the inactivation rate when compared to a single electrolysis approach in which the main inactivation mechanism consisted on the indirect oxidation in the bulk solution by means of photo-activated species derived from active chlorine (Martín de Vidales et al., 2016).

### 6.2 Fundamentals of photo-electrocoagulation

Photo-assisted electrocoagulation in another indirect disinfection process that has attracted the attention of several research groups around the world. As depicted in Fig. 3, in electrocoagulation (EC) processes, electrical current through the cell causes not only the electrochemical dissolution of the anode, but also the formation of a coagulant which in turn separates the contaminant from the solution (Çalışkan et al., 2020). In EC reactors M^n+^ is anodically released on-site and OH^−^ ions in solution produce metal monomeric and polymeric hydroxide complex species (e.g. M(OH)_n_) which act as coagulant agents that catch and destabilize colloidal species in wastewater (Çalışkan et al., 2020). Additionally, the H_2_ gas bubbles generated at the cathode cause the flotation of pollutants and, consequently, an electro-flotation phenomenon can take place (Bruguera-Casamada et al., 2019). With the integration of a UV energy source, photo-electrocoagulation processes emerge as an integrated approach in which the electro-dissolution of Fe or Al anodes is used to generate coagulant particles that not only remove suspended solids, colloidal material, pathogens as well as other dissolved solids in contaminated water (Eq. (24) – (25)), but simultaneously promotes the formation of disinfecting ^•^OH and chlorine radicals by means of UV or visible radiation (Eq. (26) – (27)) (Cotillas et al, 2014a, 2014b; Garcia-Segura and Brillas, 2017).

(24) $$\text{Fe}+2{\text{H}}_{2}\text{O}\to {\text{Fe}\left(\text{OH}\right)}_{2\left(\text{S}\right)}+{\text{H}}_{2}$$

(25) $$\text{Al}+3{\text{H}}_{2}\text{O}\to {\text{Al}\left(\text{OH}\right)}_{3\left(\text{S}\right)}+\frac{3}{2}{\text{H}}_{2}$$

(26) $${\text{ClO}}^{-}+hv\to {\text{O}}^{•-}+{\text{Cl}}^{•}$$

(27) $${\text{O}}^{•-}+{\text{H}}_{2}\text{O}\to {\text{OH}}^{-}+•\text{OH}$$

#### 6.2.1 Pathogen inactivation or removal using photo-electrocoagulation

Photo-electrocoagulation is an interesting approach that has been shown to be an attractive alternative for the treatment of actual wastewater from secondary settles using either Al or Fe anodes and SS cathode (Cotillas et al, 2014a, 2014b). The photo-assisted process allowed to obtain a substantial increase in the production of Fe coagulants with the related decrease in turbidity at low current intensities, when compared to the single EC approach. Furthermore, the incorporation of UV irradiation to the disinfection system results in a substantially smaller current density requirement (1.44 A/m^2^) for complete inactivation of *E. Coli* when compared to the non-illuminated process. The main disinfectant species formed during photo-electrocoagulation for both, Al and Fe anodes, are hypochlorite and chloramines. The difference is assumed to be due to the photo-induced production of ^•^OH and chlorine radicals that result from hypochlorite decomposition (Eq. (26) – (27)). In this regard, it is important to point out that the application of high current densities during the photo-electrocoagulation of urban wastewater samples reduces the process efficiency due to an increase in the solid’s concentration, which in turn induces a decrease in the UV transmission of solution (Cotillas et al, 2014a, 2014b). Despite the fact that both Al and Fe anodes are efficient for *E. coli* inactivation, it is also interesting to note that while the electrode consumption of the Fe is 1.7 times larger than that for an Al electrode (1 *vs.* 0.59 kg/m^3^), Al anodes need three times more energy than that required for Fe electrodes (17.4 *vs*. 5 kWh/m^3^) (Çalışkan et al., 2020).

Lalwani et al. (2019) on the other hand, studied the sequential two step electrocoagulation-photo-catalytic oxidation processes for the treatment of a pharmaceutical industry effluent in which a system using either Al or Fe electrodes, achieves complete elimination of a pre-existing microbial population of *E. coli* in a crude drug effluent (Lalwani et al., 2019).

Additional synergistic effects for the purpose of disinfection, have been observed by adding ozone to the photo-electrocoagulation treatment system (Barzegar et al., 2019). In this case, UV irradiation in the presence of O_3_ increases the photo-electrocoagulation process efficiency by means of additional free radicals which are produced either by direct photo-activation of O_3_ (Eq. (28) and (29)) or by the photo-decomposition of iron hydroxide species (see Eq. (33)) (Barzegar et al., 2019).

(28) O3+UV→O(D1)+O2

(29) O(D1)+H2O→•OH+•OH

### 6.3 Fundamentals of cathodic processes

In addition to the pathogen inactivation processes promoted in the anodic side of a photo-assisted electrolytic process, there are also complementary disinfection events taking place in the cathodic region. The electrocatalytic reduction of dissolved oxygen via 2e^−^ at cathode surface for example, produces H_2_O_2_ in acidic medium according to Eq. (30) (Brillas, 2020; Brillas et al., 2009; Peralta-Hernández et al., 2006).

(30) $${\text{O}}_{2}+2{\text{H}}^{+}+2{\text{e}}^{-}\to {\text{H}}_{2}{\text{O}}_{2}$$

For this purpose, electrodes made of carbonaceous materials such as carbon fibers, carbon felts and reticulated carbon vitreous are commonly used due to their high superficial area, high overpotential for H_2_ evolution and to their low catalytic activity for H_2_O_2_ reduction (Bañuelos et al., 2016; García-Rodríguez et al., 2016; Mousset and Dionysiou, 2020; Pérez et al., 2017a; Zárate-Guzmán et al., 2019; Zhou et al., 2018). Furthermore, the performance of the carbonaceous cathodes towards H_2_O_2_ generation can be improved by the addition of PTFE and carbon black (Jiao et al., 2020; Pérez et al, 2017b, 2019; Zhang et al., 2020c; Zhao et al., 2019).

The electrochemical generation of H_2_O_2_ in the presence of Fe^2+^ results in the Fenton mixture which readily produces ^•^OH radicals (Eq. (31)). This electrochemical approach is commonly known as the electro-Fenton advanced oxidation process and due to its high oxidation power and efficiency, it has been intensively explored for different water treatment applications (Dirany et al., 2012; Fernández et al., 2018; García-Espinoza et al., 2019; Robles et al., 2020a; Sirés et al., 2014). The continuous ^•^OH production in this case, is favored through the concomitant cathodic reduction of Fe^3+^ to Fe^2+^ as shown in Eq. (32) (Deng et al., 2020; Thiam et al., 2020).

(31) $${\text{Fe}}^{2+}+{\text{H}}_{2}{\text{O}}_{2}\to •\text{OH}+{\text{OH}}^{-}+{\text{Fe}}^{3+}$$

(32) $${\text{Fe}}^{3+}+{\text{e}}^{-}\to {\text{Fe}}^{2+}$$

The performance of the electro-Fenton process can further be improved by irradiation with UV or visible energy from either a commercial lamp emitting UVA with λ = 315–400 nm, UVB with λ = 280–315 nm or UVC with λ = 100–280 nm or using natural sunlight in (Brillas, 2020; Brillas et al., 2000; Coria et al., 2018; Thiam et al., 2020). The radiation energy causes the photo-reduction shown in Eq. (33) that transforms the Fe^3+^ species at acidic pH into Fe^2+^, as well as the photolysis of some refractory intermediates. A typical example of this phenomenon is the photo-decarboxylation of stable complexes of linear carboxylic acids with Fe^3+^ (Eq. (34)) (Brillas, 2020; Coria et al., 2018; Thiam et al., 2020).

(33) $${\text{Fe}\left(\text{OH}\right)}^{2+}+hv\to {\text{Fe}}^{2+}+•\text{OH}$$

(34) $${\text{Fe}\left(\text{OOCR}\right)}^{2+}+hv\to {\text{Fe}}^{2+}+{\text{CO}}_{2}+{\text{R}}^{•}$$

Under high energy irradiation of UVC energy, additional photolysis events of organic intermediates as well as homolysis of H_2_O_2_ to produce ^•^OH radicals via Eq. (35) also occurs.

(35) $${\text{H}}_{2}{\text{O}}_{2}+hv\to 2•\text{OH}$$

#### 6.3.1 Reports of photo-assisted electrochemical reduction

The photo-assisted processes described are not only used to treat organic pollutants in water effluents but have also been explored for the development of disinfection technology. In this way, pathogen inactivation based on an electrochemical reduction processes has been tested as an alternative to improve the efficiency of solar disinfection, using a reticulated vitreous carbon cathode and a Ti/RuO_2_ anode (Jin et al, 2020a, 2020b). The preparatory anodization of the cathode allowed to duplicate the amount of electrogenerated H_2_O_2_ due to the addition oxygen-bearing functional groups on the electrode surface. Combining H_2_O_2_ electro-generation with sunlight shortened the *E. coli* disinfection time from 150 to 120 min (Jin et al., 2020a). It is also interesting to note that the authors of this study found that the increase in the applied current density resulted in larger amounts of electrochemically produced H_2_O_2_ which surprisingly had no effect on the disinfection (Jin et al., 2020b). Pathogen inactivation by the photo-electrochemical reduction is therefore carried out by the indirect action of photons and electrochemically produced ROS such as H_2_O_2_ and singlet oxygen species (Jin et al., 2020b).

#### 6.3.2 Reports of photo-assisted electro-Fenton

The indirect oxidation by the Fenton reaction assisted by UV irradiation has also been tested for disinfection purposes. (Peralta-Hernández and Godínez, 2014) for example, used a tubular photo-reactor where bare and TiO_2_-covered carbon cloths were used as cathode and anode respectively. Using electrochemically produced H_2_O_2_ and Fe^2+^ ions previously dispersed on a cation exchange membrane, they achieved full inactivation of *E. coli* after 10 min of treatment (Peralta-Hernández and Godínez, 2014). The photo-electro Fenton process is also efficient for the elimination of the antibiotic activity of antibiotics against microorganisms such has *S. aureus* and *E. coli*, in a reactor constructed with a gas diffusion electrode as cathode, a Ti/IrO_2_ or BDD as anode, UV irradiation and dissolved Fe^2+^ (Valero et al., 2017; Vidal et al., 2019; Villegas-Guzman et al., 2017). Compared with electro-Fenton, the H_2_O_2_ accumulation observed in photo-electro Fenton processes is significantly smaller. This observation can be explained by considering (i) the photo-decomposition of H_2_O_2_ during the photo-electro Fenton process (Eq. (35)) and (ii) the light promoted increase of the Fenton reaction (Eq. (31)), due to the reconversion of Fe^3+^ into Fe^2+^ (Eq. (32)) (Villegas-Guzman et al., 2017). In this way, the photo-electro Fenton process allows a more efficient elimination of antimicrobial activity when compared to other electrochemical processes such as the electrochemical oxidation and the electro-Fenton approach due to a synergistic effect between light and the Fenton mixture that noticeably increases the production of ROS (Bruguera-Casamada et al., 2019; Valero et al., 2017; Vidal et al., 2019).

It is also interesting to point out that photo-assisted EAOPs can be used as a first stage of treatment, aimed to increase the biodegradability of the pollutants before the biological process is applied. For example, Vidal et al. (2018) reported a study of the performance of a photo-electro Fenton process, using a carbon-PTFE air-diffusion electrode as cathode, a BDD anode and UV irradiation, followed by anaerobic biodigestion (Vidal et al., 2018). Using this combination, the antimicrobial activity for *S. aureus* revealed complete inactivation (Vidal et al., 2018). Furthermore, it was possible to observe that the integration of a biological system with a photo-electrochemical processes, allowed the removal of recalcitrant intermediate compounds which are commonly formed at the end of the first electrochemical treatment stage.

EC processes with Fe electrodes followed by UVA irradiated photo-electro-Fenton processes that employ an air-diffusion cathode and a BDD or DSA anode, have also been tested for the disinfection of wastewater (Bruguera-Casamada et al., 2019). In this type of processes, the bacteria were poorly removed by the flocs formed in EC but largely inactivated by the photo-electro Fenton process in which the BDD anode promotes pathogen inactivation at circumneutral pH by active chlorine and BDD(^•^OH) radicals, whereas the DSA led to quick inactivation at pH 3.0 (Bruguera-Casamada et al., 2019).

As depicted in Fig. 4a, the indirect inactivation of pathogens can be carried out by photo-assisted EOPs such as photo-coagulation, photo-electro-Fenton and by electrogenerated ROS, RHS and sulphate radical species (letters G, H, I, J and K inf Fig. 4a). Such mechanism has been proved in the disinfection of contaminated water with *E. coli*, adenovirus, *P. aeruginosa*, bacillus species, *M. aeruginosa* and *C. parapsilosis*. It is therefore interesting to point out that the combination and the synergistic effect of direct, quasi-direct and indirect oxidation mechanisms have also been explored in the literature (see Fig. 4a, letters B, C, D and F) for the inactivation of microorganisms.

## 7 Economic aspects and scale-up of the photo-assisted EAOPs

The economic cost of photo-assisted EAOPs for water disinfection could be divided in the initial investment (which is mainly related to electrodes and lamps) and the operating costs, which are associated to energy requirements and electrode and parts replacement. Typical anode materials such as BDD and DSA are known to be expensive, but very stable, thus providing low replacement and maintenance costs. Semiconductor, carbonaceous materials, and metallic electrodes such as Al or Fe on the other hand, are affordable options for EAOPs but have the associated disadvantage of a higher replacement frequency. In terms of energy requirements for pathogen inactivation promoted by EAOPs, the specific electric energy (E_EO_) in kWh m^−3^ order^−1^, can be calculated using Eq. (36), where *E* is the cell voltage (V), W_lamp_ the power of the lamp (kW), *I* the current intensity (kA), *t* the operation time (h), *V* the volume (m^3^) and C_0_ and C_f_ the initial and final pathogen concentration after 1 order of magnitude removal (CFU mL^−1^), respectively (Herraiz-Carboné et al., 2020b).

(36) $${\text{E}}_{\text{EO}}=\frac{\left(\text{E I}+{\text{W}}_{\text{lamp}}\right)\text{t}}{\text{V Log}\frac{{\text{C}}_{0}}{{\text{C}}_{\text{f}}}}$$

(Herraiz-Carboné et al., 2020b) reported that the electric energy consumption for UV assisted electrochemical oxidation using a BDD anode at the high applied current density of 50 A m^−2^, is slightly higher than that required for the UV disinfection process (0.395 *vs*. 0.389 kWh m^−3^ order^−1^). Nevertheless, the use of a MMO anode and BDD anode at the low current density of 5 A m^−2^, leads to lower electric energy requirements. In this way, photo-assisted EAOPs may successfully compete with other AOPs in terms of sustainability, for instance, photo-conductive diamond electrochemical oxidation shows the faster and more efficient performance in terms of energy consumption when compared to the same process when assisted by ultrasound and with the process itself at bench scale remediation plants (Fernández-Marchante et al., 2021a). To obtain the economic viability of the photo-assisted EAOPs for water disinfection however, it is important to consider the use of renewable energy sources and to elucidate the optimal operational conditions of such processes; thus allowing safe treated water produced under minimum energetic requirements.

Reports on photo-assisted EAOPs for water disinfection at pilot or real wastewater plant scale, are scarce in the literature. This not only reflects intellectual property and know-how considerations, but also the incipient nature of this approach. In any case however, the scale-up of EAOPs has been reported in some investigations. For example, Cotillas et al. (2020), recently reported wastewater reclamation in an integrated electro-disinfection-electrocoagulation process (Cotillas et al., 2020). Such approach, operates with a real secondary effluent and a flow rate of 50 L h^−1^; allowing complete *E. coli* depletion at current densities in the 5–10 A m^−2^ range. Otter et al. (2020) on the other hand, evaluated water supply systems operating with solar-driven electro-chlorination processes in rural regions (Otter et al., 2020). This technology manages to supply communities with safe water. Isidro et al. (2018) also evaluated a system integrated by coagulation-flocculation, lamellar sedimentation and filtration processes in a column unit at pilot scale. This process was followed by electrochemical disinfection using a commercial CabECO® cell reaching more than 4 log units of disinfection of highly faecal-polluted surface water (Isidro et al., 2018)*.*

## 8 Future trends and perspectives

Since the high energetic consumption is one of the major drawbacks in electrochemical processes, it is important the development of renewable energy –driven photo-assisted EAOPs technologies to minimize the energetic cost and to be potentially implemented in remote locations (Ganiyu et al., 2020; Ganiyu and Martínez-Huitle, 2020). Among the different approaches under study, the conversion of solar energy through the use of photo-voltaic cells (Ganiyu et al., 2019; Zhang et al., 2016), wind energy by wind turbines (Fernández-Marchante et al., 2021b; Malek et al., 2016) and the use microbial fuel cells (Hou et al., 2020; Jiménez González et al., 2020) represent attractive directions for the development of technologies to reduce the environmental problems associated with fossil fuels.

On the other hand, it is also imperative the develop new electrode materials. Sub-stoichiometric titanium oxides (Ti_n_O_2n-1_, 4 ≤ n ≤ 10, also known as Magneli phases) for example, are ceramic and promising anode materials for the disinfection and treatment of water (Becerril-Estrada et al., 2020; Ganiyu et al., 2016; Ouarda et al., 2020). Particularly, Ti_4_O_7_ materials are attractive electrodes due to their high electrical conductivity (∼1000 S cm^−1^), chemical stability, high oxygen evolution potential (2.0–2.5 V *vs*. SHE) and lower manufacturing cost when compared to non-active anodes such as BDD (Silva Barni et al., 2020; Trellu et al., 2020). The degradation pathway using Ti_4_O_7_ anodes is associated by “quasi-free” M(^•^OH) electrogenerated radicals (Olvera-Vargas et al., 2018). To our knowledge, there are not studies on the evaluation of Ti_4_O_7_ as an anode material in a photo-assisted EAOPs for water disinfection purposes. Biochar also constitute excellent materials for the promotion of H_2_O_2_ by means of the reduction of dissolved oxygen. These materials are obtained by spend biomass sources such as animal feedings (Y. Wang et al., 2019), agricultural or woody materials (Deng et al., 2019), food wastes (Tovar et al., 2019) and sewage sludge (Huang et al., 2020; Mian and Liu, 2019) among others (Do Minh et al., 2020). Recent studies of biochar materials focuses on their use as simultaneous adsorbents and 3D-cathodes where adsorption, disinfection and pollutants degradation occur followed by the regeneration of the electrode material surface (Bañuelos et al., 2015; Mesones et al., 2020; Robles et al., 2020b). The integration of such materials in photo-assisted EAOPs for the inactivation of microorganisms by indirect oxidation will be a relevant topic of research and technology development in upcoming years.

Moreover, since most of the studies of the photo-assisted EAOPs have focused on the inactivation of *E. coli*, it is necessary to extend the pathogen inactivation spectrum of these technologies by evaluating other microorganisms such as helminth eggs, antibiotic-resistant bacteria and viruses. It is important to increase the number of studies that focus on the inactivation of the particularly resistant helminth eggs since around 2.5 billion people worldwide are infected with helminths (Jiménez et al., 2016). In view of the membrane resistant character of these pathogens, recent studies have shown promising results by approaching the problem through AOPs (Morales-Pérez et al., 2016; Robles et al., 2020a). Although research has also been carried out on the inactivation of some of antibiotic-resistant bacteria, the validation of the photo-assisted EAOPs on them (*E. coli, K. pneumoniae. S. aureus, S. pneumoniae,* Nontyphoidal *salmonella, Shigella* species and *N. gonorrhoeae)* is important since the WHO has classified bacterial resistance as one of the greatest threats to public health in the 21st century (World Health Organization, 2014).

The recent global pandemic coronavirus disease (COVID-19) for instance, has triggered a Public Health Emergency of International Concern. Even though the known route of transmission is due to direct contact or via respiratory droplets, recent studies have reported that SARS-CoV-2 ribonucleic acid has been found in WWTP samples (La Rosa et al., 2020; Mandal et al., 2020). In fact, sodium hypochlorite treatment and the current disinfection guideline outlined by the WHO might not secure a complete removal of SARS-CoV-2 in medical wastewater septic tanks (Zhang et al., 2020a,b,c,d,e). In the context of a fast-changing understanding of the COVID-19 disease and the SARS-CoV-2 virus, it is essential to develop effective water disinfection processes that guarantee the complete inactivation of the virus. Since photo-assisted EAOPs have been successfully demonstrated as affective alternative for the inactivation of a variety viruses (Li et al., 2014), such processes stand out as a potentially important approach for the treatment of SARS-CoV-2 contaminated wastewater.

Furthermore, efforts need to focus on the development of mature technologies to evolve proof of concept studies into technology packages ready for commercialization (Lacasa et al., 2019). To achieve that point, it is mandatory to carry out studies in scenarios with the consideration and complications associated to real-water matrix effects, overcoming limitations of synthetic laboratory solutions which, on the one hand, typically use concentrations of pollutants that are higher than those found in real wastewater; and which are also characterized by low electric conductivity values than those commonly employed in laboratory assays (e.g., 0.05 M Na_2_SO_4_) (Garcia-Segura et al., 2020). Since the electric conductivity defines for the most part the applied current for a given potential, the increase of the conductivity decreases the energetic consumption of the processes (Garcia-Segura et al., 2018). Other constituents of the real-wastewater which are necessary to evaluate in photo-assisted EAOPs are the dissolved organic matter (DOM), the NOM and NO_3_^−^. The DOM is usually ignored in lab-scale experiments resulting in high efficacies due to the lack of the scavenger-effect of the DOM (Giannakis et al., 2021). The NOM consist of humic substances such as humic acid and fulvic acid, and they can have both inhibitory and synergistic effects on the performance of AOPs. For instance, the NOM can act as photosensitizer improving the yields of the UV/H_2_O_2_ process (Sharma et al., 2015), and they can introduce a inhibitory effect on the efficiency of the electrochemical oxidation process (Lebik-Elhadi et al., 2018). The NO_3_^−^ on the other hand, can absorb UV irradiation acting as an inner filter to hinder light transmission through the aqueous solution (Yang et al., 2019). Finally, it has been reported that NO_3_^−^ could generate ^•^OH radicals in aquatic media under UV irradiation, thus increasing the efficiency of the AOPs (Spiliotopoulou et al., 2015). As it can be noted, the effect of these substances on the AOPs is still not clear and further studies on the subject are needed. Future studies can also help to fill the herein pointed-out gaps, thus facilitating the application of photo-assisted EAOPs for water disinfection purposes in real scenarios.

## 9 Conclusions

Photo-assisted EAOPs have emerged as effective methods to achieve the disinfection of water, since they combine the electro-generation of reactive species at electrodes and the photolytic production of radicals. This review presented an overview of the oxidation mechanisms by photo-assisted EAOPs for water disinfection, in which the place where the pathogen’s inactivation occurs, defines to a good extent the oxidation mechanism. The quasi-direct oxidation by ^•^OH was found as the most studied mechanism of pathogen inactivation mostly generated by photo-electrocatalysis using photo-anodes, by photo-assisted electrochemical oxidation using DSA and BDD anodes and by photo-electro-Fenton processes. The presence of halides in solution results in the photo-electro-generation of RHS, which in turn, promotes the indirect oxidation of pathogens.

Photo-assisted indirect oxidation processes like electrocoagulation are also efficient processes for microorganism inactivation. Photo-assisted electrogenerated persulphate process for water disinfection is nowadays scarcely reported and an interesting option to be explored in subsequent years.

Among the microorganisms that have been explored, *E. coli* is by far the most studied pathogen and therefore, future efforts should focus on the evaluation of other antibiotic-resistant bacteria as well as resistant pathogens like helminth eggs and highly toxic and health-threaten agents such as viruses.

UV irradiation on the other hand, is the most popular photon’s source in photo-assisted EAOPs studies but, it is important for the development of cost optimized disinfection processes that, new materials, arrangements and methods are explored in the future in order to take advantages of solar energy.

Aiming to achieve the deployment of these processes at pilot and full-scale, the understanding of the pathogen inactivation mechanism, the evaluation of their performance using real-wastewater matrixes, the optimization of the electrochemical reactors’ configuration as well as renewable energy–driven technologies are needed.

## Declaration of competing interest

The authors declare that they have no known competing financial interests or personal relationships that could have appeared to influence the work reported in this paper.
