# Supplementary material for: Photo-assisted electrochemical advanced oxidation processes for the disinfection of aqueous solutions: A review
Source: Chemosphere. 2021 Jul;274:129957. doi: 10.1016/j.chemosphere.2021.129957 (PMC8121763; doi:10.1016/j.chemosphere.2021.129957)
Supplement: Multimedia component 1 [file mmc1.docx]

**Supplementary material**

**Photo-assisted electrochemical advanced oxidation processes for the disinfection of aqueous solutions: A review**

Josué Daniel García-Espinoza^1^, Irma Robles^1^, Alfonso Durán-Moreno^2^, Luis A. Godínez^1,^ *

^1^ Centro de Investigación y Desarrollo Tecnológico en Electroquímica, Parque Tecnológico Querétaro Sanfandila, 76703, Pedro Escobedo, Querétaro, México.

^2^ Facultad de Química, Universidad Nacional Autónoma de México, CDMX, México.

* Corresponding author: Luis A. Godínez, lgodinez@cideteq.mx

Fig. SM1. (a) Percentages of scientific reports classified as direct, quasi-direct and indirect oxidation processes in photo-assisted EAOPs for water disinfection; (b) Percentages of scientific reports classified by type of irradiation in photo-assisted EAOPs for water disinfection.
